# Outcome and Safety of Genicular Artery Embolization for Knee Osteoarthritis: A Systematic Review and Meta-Analysis

**DOI:** 10.1007/s00270-026-04502-7

**Published:** 2026-06-16

**Authors:** Arian Taheri Amin, Isaac Eli Martínez Safar, Ricardo Baquero Trujillo, Juan Guillermo Trujillo Buritica, Osman Ahmed, Kai Jannusch, Peter Minko

**Affiliations:** 1 Department of Diagnostic and Interventional Radiology, Charité Universitätsmedizin Berlin, Corporate Member of Freie Universität Berlin and Humboldt-Universität Zu Berlin, Berlin, Germany; 2 Department of Diagnostic and Interventional Radiology, Medical Faculty, University Hospital Duesseldorf, Campus Virchow-Klinikum (CVK), Augustenburger Platz 1, D-40225 Duesseldorf, Germany; 3 School of Medicine, Faculty of Health Sciences, Universidad Libre Seccional Barranquilla, Barranquilla, Colombia; 4 Department of Radiology and Diagnostic Imaging, Clínica Reina Sofía, Bogotá, Colombia; 5 Joint and Vascular Institute, Libertyville, IL USA; 6 Department of Radiology, University of Utah, Salt Lake City, IL USA; 7 Department of Diagnostic and Interventional Radiology, Charité Universitätsmedizin Berlin, Campus Virchow-Klinikum (CVK), Augustenburger Platz 1, 13353 Berlin, Germany

**Keywords:** Genicular artery embolization, Knee osteoarthritis, Meta-analysis, Systematic review, Pain, Minimally invasive treatment

## Abstract

This systematic review and meta-analysis evaluated pain and functional outcomes, safety, and differences between embolic agents in genicular artery embolization (GAE) for knee osteoarthritis. Forty-five studies including 2205 patients were analyzed. Across predominantly uncontrolled studies, clinically relevant improvements were observed, with pooled Visual Analogue Scale reductions of − 37.5 points at 1 month and − 37.1 at 12 months. Western Ontario and McMaster Universities Osteoarthritis Index improved by − 28.9 at 1 month, and Knee Injury and Osteoarthritis Outcome Score-Pain by + 23.6 at 6 months. Mean changes exceeded clinically important thresholds. No significant subgroup interaction between permanent and temporary embolic agents was observed. The most frequent adverse events were transient skin discoloration (11%) and hematoma (3%). Overall, the certainty of evidence ranged from very low to low.

**Graphical Abstract**

**Supplementary Information:**

The online version contains supplementary material available at https://doi.org/10.1007/s00270-026-04502-7.

**Figure:**
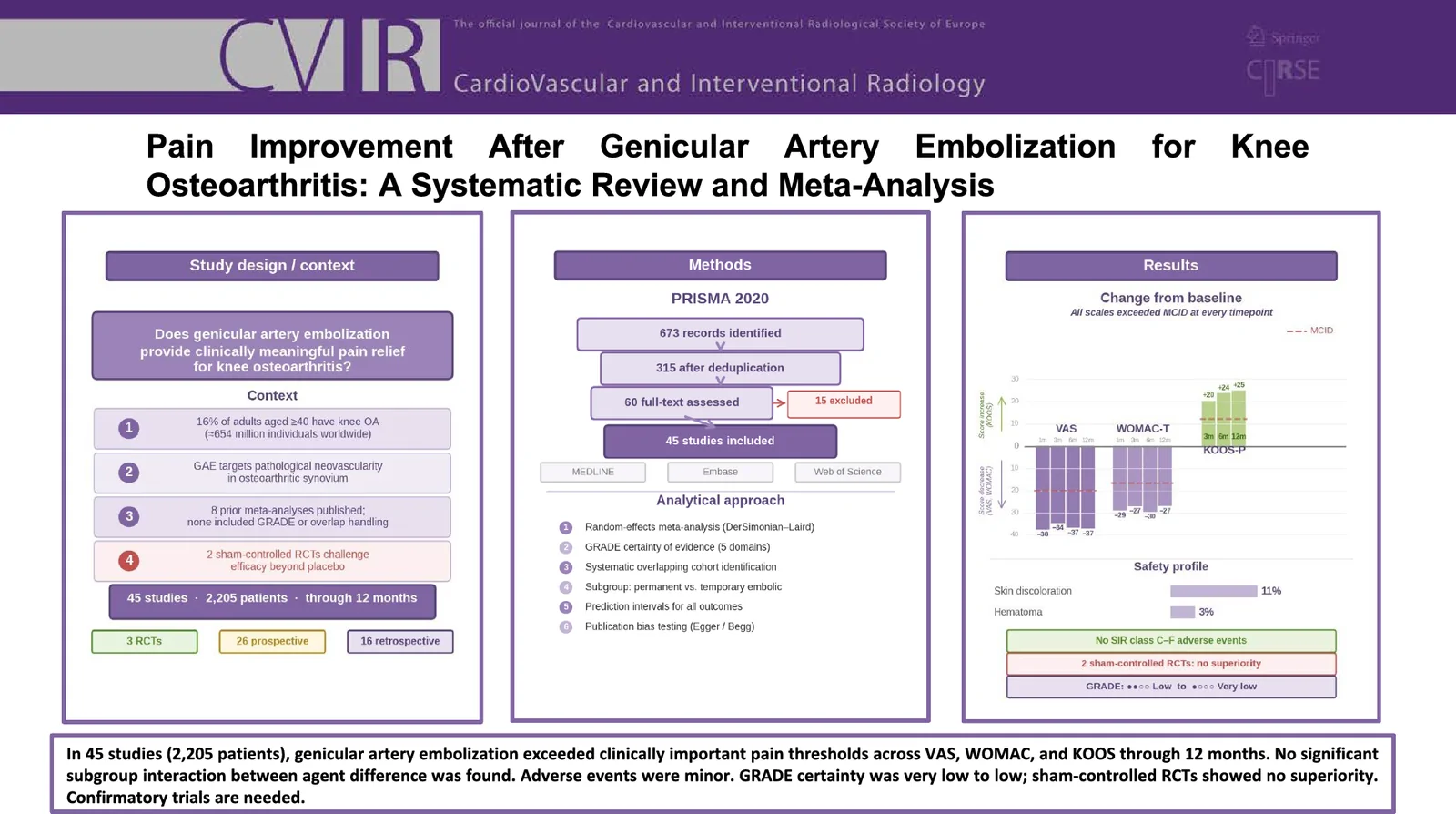

## Introduction

Approximately 16% of the global population aged 40 years and older is affected by knee osteoarthritis (KOA), corresponding to an estimated 654 million individuals in 2020 [1, 2]. While current guidelines recommend a stepwise approach including exercise and pharmacotherapy to total knee arthroplasty for end-stage disease [3, 4], a substantial proportion of patients fall into a therapeutic gap between exhausted conservative options and surgical candidacy. By targeting aberrant neovascularization and the accompanying sensory nerve fiber ingrowth within the inflamed synovium, genicular artery embolization (GAE) aims to interrupt the cycle of synovitis-driven pain [5]. First reported in 2015 [5], GAE has since been evaluated in prospective cohorts, sham-controlled randomized trials and retrospective studies across 15 countries [6–8].

Several meta-analyses have pooled GAE outcomes since 2020. Early reviews were limited to 3–11 studies and fewer than 270 patients [9–13]. The largest analysis to date, by Chlorogiannis et al. [13], included 21 studies with 633 patients and reported visual analogue scale (VAS) reductions of 38–41 points across all timepoints, with I^2^ values of 57–93%. Whether these pooled estimates remain stable with a substantially expanded evidence base that now includes three sham-controlled randomized controlled trials (RCTs) [14–16], the largest single-center cohort (n = 333) [17], and novel embolic agents [18, 19] remains unclear. None of the prior analyses applied formal certainty-of-evidence grading, calculated prediction intervals, or addressed the growing problem of overlapping cohorts from high-volume centers.

The purpose of this systematic review and meta-analysis is to (a) pool pain and functional outcomes after GAE across VAS, Western Ontario and McMaster Universities Osteoarthritis Index (WOMAC), and Knee Injury and Osteoarthritis Outcome Score (KOOS) at one to twelve months; (b) assess the safety of GAE; (c) compare permanent versus temporary embolic agents; (d) systematically identify and handle overlapping institutional cohorts; and (e) grade the certainty of evidence.

## Materials and Methods

### Study Design, Search Strategy and Eligibility

This review followed Preferred Reporting Items for Systematic Reviews and Meta-Analyses (PRISMA) 2020 guidelines [20]; the flow diagram was generated using the PRISMA2020 R package [21] and was registered with the Open Science Framework. MEDLINE (PubMed), Embase, and Web of Science were searched from inception through February 2026. The search combined MeSH terms and free-text keywords: “genicular artery,” “embolization/embolisation,” “osteoarthritis,” “knee,” and synonyms (full strategy in Supplementary Table S1). Reference lists of included studies and relevant reviews were manually screened.

Eligible studies enrolled ≥ 6 adult patients (≥ 18 years) with symptomatic KOA of any Kellgren–Lawrence (K-L) grade who underwent GAE with any embolic material and reported at least one validated pain or functional outcome (VAS, WOMAC, KOOS). RCTs, prospective and retrospective studies were included. Case reports, conference abstracts without full text, reviews, protocols, animal studies, non-English-language studies, and GAE in non-OA indications were excluded.

### Study Selection and Data Extraction

Two reviewers independently screened titles/abstracts and full texts (O.A; I.E.M.S). Data were extracted using a standardized form. When outcomes were reported only graphically, WebPlotDigitizer (Version 4.7) was used to digitize data from plots and graphs in images. VAS scores on a 0–10 scale were converted to 0–100. Where outcomes were reported as median and interquartile range (IQR), means and standard deviations (SDs) were estimated using the methods of Wan et al. [22] and Luo et al. [23]. Studies reporting IQR = 0 at baseline were excluded from pooling for that outcome.

### Overlapping Cohorts

Overlapping cohorts were identified by cross-referencing author statements, trial registrations, and institutional affiliations with overlapping enrollment periods. Seven institutional groups were identified [5, 7, 8, 24–35], and two probable overlapping groups [15, 36–42]. The most comprehensive publication served as the primary source. Earlier publications were retained only for specific outcome-timepoint combinations not reported elsewhere. Full decision diagrams are detailed in Supplementary Table S7.

### Risk of Bias and Statistical Analysis

Risk of bias was assessed using the Cochrane RoB 2 tool for RCTs [43] and ROBINS-I for non-randomized studies [44]. Random-effects meta-analysis was performed using the DerSimonian–Laird estimator [45]. The effect measure was the weighted mean difference (WMD) relative to baseline. Pooled adverse event proportions were estimated using the Freeman–Tukey double arcsine transformation. Prediction intervals were calculated for all estimates with k ≥ 3. Pre-specified subgroup analyses compared permanent versus temporary embolic agents, with a display rule requiring k ≥ 2 in both arms. Publication bias was assessed with funnel plots, Egger regression, and Begg rank correlation for outcomes with k ≥ 10. Sensitivity analyses included leave-one-out, restriction to low/moderate risk-of-bias studies, and sequential exclusion of probable overlapping cohorts. Clinical significance was evaluated using published minimal clinically important difference (MCID) thresholds: VAS reduction ≥ 20 points [46], WOMAC-Total reduction ≥ 16 points [26], and KOOS-Pain improvement ≥ 10 points [24]. All analyses were performed using R (Version 4.5.2). Percentage reductions (VAS, WOMAC) and improvements (KOOS) reported in the Results section were calculated as the pooled weighted mean difference (WMD) divided by the weighted mean baseline value across contributing studies for each outcome and timepoint, multiplied by 100. Certainty of evidence was graded using the Grading of Recommendations, Assessment, Development and Evaluations (GRADE) approach.

## Results

### Study Selection and Characteristics

**Fig. 1:**
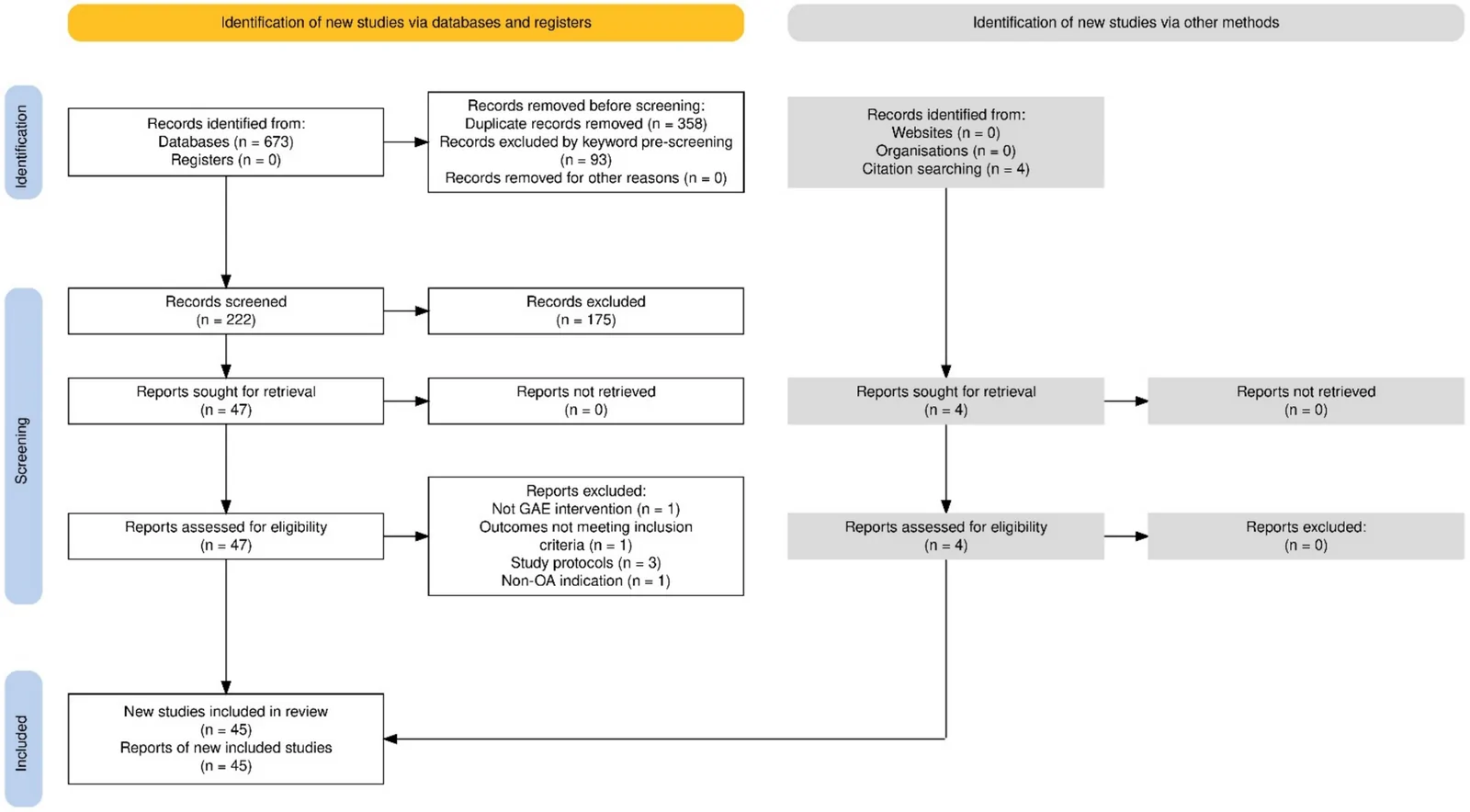
PRISMA 2020 flow diagram

From 673 database records, 315 unique records remained after deduplication (Fig. 1). After screening, 47 proceeded to full-text review alongside 4 studies from reference checking. Of 51 full texts assessed, 6 were excluded. In total, 45 studies enrolling 2,205 patients were included (Table 1).

**Table 1:** Characteristics of included studies

Study	Study design	Sample size	Knees treated	Age (years)	Female, n (%)	Male, n (%)	K-L grade	Embolic agent	Pain tools	FU (mo)
Okuno 2017 [8]	Prospective	72	95	64	49 (68)	23 (32)	1–3	IPM-CS*	VAS, WOMAC	48
Lee 2019 [47]	Retrospective	41	71	67	29 (71)	12 (29)	1–4	IPM-CS	VAS	10
Bagla 2020 [6]	Prospective	20	20	59	11 (55)	9 (45)	1–3	Embozene	VAS, WOMAC	6
Choi 2020 [48]	Retrospective	18	28	69	14 (78)	4 (22)	1–4	IPM-CS	VAS	3
Landers 2020‡ [36]	Prospective	10	10	62ᵐ	4 (40)	6 (60)	1–2	PVA / IPM-CS	VAS, KOOS	24
Padia 2021‡ [7]	Prospective	40	40	69ᵐ	31 (77)	9 (23)	2–4	Embozene	VAS, WOMAC	12
van Zadelhoff 2021 [49]	Retrospective	54	54	69ᵐ	42 (78)	12 (22)	1–4	IPM-CS	WOMAC	6
Bagla 2022 [16]	RCT	21	21	64	18 (86)	3 (14)	1–3	Absorbable MS	VAS, WOMAC	12
Russu 2022† [50]	Prospective	17	17	64	12 (71)	5 (29)	NR	IPM-CS	WOMAC	12
Bhatia 2023 [51]	Retrospective	14	20	73	11 (79)	3 (21)	2–4	ES / IPM-CS	WOMAC	24
Gill 2023 [37]	Prospective	33	33	58ᵐ	19 (58)	14 (42)	2–4	IPM-CS	KOOS	6
Landers 2023 [15]	RCT	59	59	60	37 (63)	22 (37)	2	IPM-CS	KOOS	12
Min 2023 [52]	Retrospective	71	97	64	54 (76)	17 (24)	NR	Gelatin sponge	VAS	6
Sapoval 2023 [25]	Prospective	22	23	66	13 (59)	9 (41)	3–4	Lipiodol	VAS, WOMAC	3
Shaker 2023 [53]	Prospective	16	16	50	12 (75)	4 (25)	1–3	Permanent MS	VAS, WOMAC	6
Taslakian 2023‡ [26]	Prospective	16	16	67ᵐ	6 (37)	10 (63)	2–4	Embozene	VAS, WOMAC, KOOS	12
Wang 2023 [54]	Prospective	22	24	64ᵐ	15 (68)	7 (32)	1–3	IPM-CS	VAS, WOMAC	6
Cusumano 2024 [27]	Prospective	40	40	66	31 (77)	9 (23)	2–4	Embozene	WOMAC	24
Dablan 2024‡ [38]	Retrospective	33	35	59	24 (73)	9 (27)	2–4	IPM-CS	VAS, WOMAC	3
Gorsi 2024 [55]	Prospective	10	10	56	8 (80)	2 (20)	1–3	Embosphere	VAS, WOMAC	3
Guzelbey 2024a‡ [39]	Retrospective	42	42	65ᵐ	23 (55)	19 (45)	2–4	IPM-CS	VAS, WOMAC	6
Guzelbey 2024b‡ [40]	Retrospective	79	79	65ᵐ	61 (77)	18 (23)	1–4	IPM-CS	WOMAC°	6
Hindsø 2024b‡ [28]	Prospective	17	17	56ᵐ	9 (53)	8 (47)	1–3	Embosphere	—	6
Hindsø 2024a [29]	Prospective	17	17	56ᵐ	9 (53)	8 (47)	1–3	Embosphere	VAS, WOMAC, KOOS	6
Kilickesmez 2024 [41]	Retrospective	60	60	64ᵐ	53 (88)	7 (12)	1–4	IPM-CS	VAS, WOMAC	6
Little 2024 [24]	Prospective	46	46	60ᵐ	25 (54)	21 (46)	1–3	Embosphere	VAS, WOMAC, KOOS	24
Sun 2024 [56]	Prospective	33	37	67	27 (82)	10 (18)	2–4	PVA	VAS, WOMAC	12
van Zadelhoff 2024 [14]	RCT	58	58	58	35 (60)	23 (40)	1–3	Embozene	VAS, KOOS	4
Badar 2025a [30]	Retrospective	34	42	66	27 (79)	7 (21)	2–4	PEG-MS	WOMAC	24
Badar 2025b‡ [31]	Retrospective	39	52	62	30 (77)	9 (23)	2–4	PEG-MS / EO	WOMAC	3
Badar 2025c [32]	Retrospective	68	88	65	57 (84)	11 (16)	2–4	PEG-MS / EO	WOMAC	6
Callese 2025 [57]	Retrospective	236	NR	69	NR	NR	1–4	Embozene	WOMAC	12
Dablan 2025‡ [42]	Retrospective	66	72	60	50 (76)	16 (24)	1–4	IPM-CS	VAS°	6
Fleckenstein 2025 [17]	Retrospective	333	444	68ᵐ	182 (55)	151 (45)	2–4	IPM-CS	VAS, KOOS	12
Harrison 2025‡ [58]	Prospective	30	NR	62	15 (50)	15 (50)	NR	Embosphere	VAS, KOOS	12
Little 2025 [59]	Prospective	15	15	63	12 (80)	3 (20)	1–3	SakuraBead	VAS, WOMAC	6
Sapoval 2025 [33]	Prospective	46	50	67	29 (58)	21 (42)	2–4	Lipiodol	VAS, WOMAC	12
Taheri Amin 2025a [60]	Prospective	43	NR	65ᵐ	21 (49)	22 (51)	2–4	Embosphere	VAS	12
Taheri Amin 2025c [61]	Prospective	35	35	62ᵐ	21 (60)	14 (40)	2–4	Embosphere	KOOS	6
Taheri Amin 2025b [18]	Prospective	45	45	64ᵐ	24 (53)	21 (47)	1–4	Resorbable gelatin MS	VAS, KOOS	6
Taslakian 2025 [34]	Prospective	25	25	65	13 (52)	12 (48)	2–4	Embozene	VAS, WOMAC, KOOS	12
Mabud 2026‡ [35]	Retrospective	25	25	65	13 (52)	12 (48)	2–4	Embozene	WOMAC	12
Taheri Amin 2026a [62]	Prospective	113	113	64ᵐ	56 (50)	57 (50)	1–4	Embosphere	KOOS	6
Taheri Amin 2026b [63]	Prospective	55	55	69ᵐ	32 (58)	23 (42)	4	Embosphere	VAS	12
Zolovkins 2026 [19]	Prospective	16	19	70	11 (69)	5 (31)	1–4	DSM	VAS, WOMAC	12

FU follow-up; K-L Kellgren–Lawrence; IPM-CS imipenem/cilastatin sodium; PVA polyvinyl alcohol; ES Embosphere; PEG-MS polyethylene glycol microspheres; EO ethiodized oil; DSM degradable starch microspheres; MS microspheres; mo months; NR not reported. Age reported as mean; ᵐindicates median

^*^IPM-CS used in 88/95 knees, Embosphere in 7/95. †WOMAC reported on inverted 0–100 scale (higher = better); excluded from pooled WOMAC analysis. °Binary or change-score reporting only (not pooled for absolute values). ‡Excluded from primary quantitative synthesis due to confirmed or probable cohort overlap; retained for descriptive analysis or specific outcome-timepoint combinations (see Methods and Supplementary Table S7). Letters (a, b, c) distinguish multiple publications within the same author-year group

Studies were published between 2017 and 2026 across 15 countries, with the United States (*n* = 11), Germany (*n* = 6), and Turkey (*n* = 5) contributing the most publications. Study designs included 3 RCTs, 26 prospective cohorts, and 16 retrospective studies; 42 were single-center. Median sample size was 35 (range 10–333). The largest cohort was reported by Fleckenstein et al. (*n* = 333) [17]. Mean age ranged from 50 to 73 years. A female predominance was observed (median male proportion 32%). K–L grade distribution, reported in 40 studies, most commonly included grades 2–4.

All studies required failure of prior conservative treatment. Embolic materials included temporary agents in 22 studies (imipenem/cilastatin sodium [IPM-CS] in 16, ethiodized oil emulsions in 5, others in 3) and permanent agents in 20 (Embozene in 9, Embosphere in 9, HydroPearl in 3, polyvinyl alcohol in 2); three of which compared both categories within the same cohort. Vascular access was predominantly ipsilateral via the femoral artery. Among the 33 studies reporting anesthesia type, local anesthesia alone was used in 20, moderate sedation in 12, and regional anesthesia in 1. A median of 2 to 4 genicular arteries were embolized per procedure. Technical success was defined variably as pruning of neovascularization (19 studies), resolution of hyperemic blush (9), or near-stasis of contrast (6) and reached 100% in 35 of 40 reporting studies. Follow-up ranged from 3 to 48 months.

### Overlapping Cohorts

Confirmed and probable overlapping relationships were identified across seven confirmed and two probable institutional groups (detailed in Methods and Supplementary Table S7). After applying overlap handling rules, 40 study populations contributed to quantitative synthesis after excluding 5 studies with confirmed complete overlap. Six additional studies with confirmed or probable partial overlap were retained for specific outcome-timepoint combinations where the comprehensive publication lacked extractable data.

### Risk of Bias

Among three RCTs assessed with RoB 2, Bagla 2022 [16] was at high risk (complete sham crossover at 1 month); Landers 2023 [15] and van Zadelhoff 2024 [14] were at some concerns. Both adequately blinded sham-controlled RCTs reported negative primary outcomes. All 42 non-randomized studies (ROBINS-I) were at serious or critical overall risk, with confounding rated critical in all 35 single-arm studies because of the absence of a control group (Supplementary Tables S2–S6).

### Pain Outcomes

**Fig. 2:**
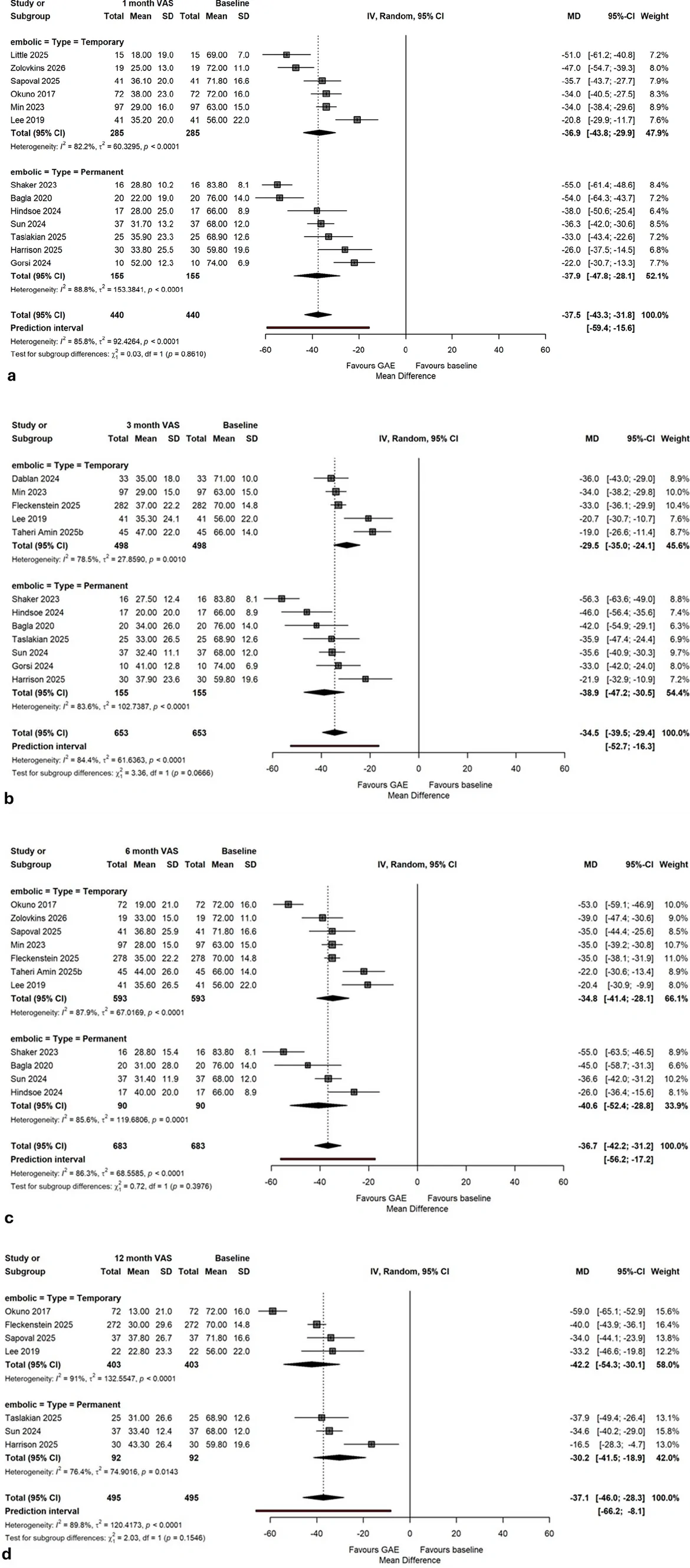
Forest plots of pooled VAS. Forest plots of pooled VAS (0–100) from baseline after genicular artery embolization at **a** 1 month (k = 13; WMD = −  37.5; I^2^ = 86%), **b** 3 months (k = 12; WMD = −  34.5; I^2^ = 84%), **c** 6 months (k = 11; WMD = −  36.7; I^2^ = 86%), and **d** 12 months (k = 7; WMD = −  37.1; I^2^ = 90%), with subgroup analysis by embolic material type (permanent vs. temporary)

GAE produced significant VAS reductions at all timepoints (Table 2; Fig. 2): WMD − 37.5 at 1 month (55% reduction; k = 13; 95% CI − 43.3 to − 31.8; I^2^ = 86%), − 34.5 at 3 months (51% reduction; k = 12; I^2^ = 84%), − 36.7 at 6 months (53% reduction; k = 11; I^2^ = 86%), and − 37.1 at 12 months (54% reduction; k = 7; 95% CI − 46.0 to − 28.3; I^2^ = 90%). All VAS reductions exceeded the MCID (reduction ≥ 20 points) [46], and all prediction intervals excluded zero.

**Table 2:** Pooled outcomes

Outcome	Time	k	N	WMD [95% CI]	I^2^ (%)	Prediction interval	GRADE
VAS	1 mo	13	440	− 37.5 [− 43.3; − 31.8]	86	[− 59.4; − 15.6]	●○○○
VAS	3 mo	12	653	− 34.5 [− 39.5; − 29.4]	84	[− 52.7; − 16.3]	●○○○
VAS	6 mo	11	683	− 36.7 [− 42.2; − 31.2]	86	[− 56.2; − 17.2]	●○○○
VAS	12 mo	7	495	− 37.1 [− 46.0; − 28.3]	90	[− 66.2; − 8.1]	●○○○
WOMAC-T	1 mo	10	312	− 28.9 [− 35.8; − 22.0]	91	[− 54.0; − 3.9]	●○○○
WOMAC-T	3 mo	9	273	− 26.9 [− 32.6; − 21.3]	80	[− 45.8; − 8.1]	●○○○
WOMAC-T	6 mo	7	262	− 29.5 [− 33.9; − 25.0]	62	[− 41.9; − 17.1]	●●○○
WOMAC-T	12 mo	3	130	− 26.8 [− 37.0; − 16.5]	88	[− 69.4; 15.9]	●○○○
WOMAC-P	1 mo	8	277	− 6.6 [− 8.3; − 5.0]	86	[− 12.1; − 1.1]	●○○○
WOMAC-P	3 mo	9	329	− 6.5 [− 7.9; − 5.2]	84	[− 11.1; − 1.9]	●○○○
WOMAC-P	6 mo	6	246	− 6.9 [− 8.5; − 5.2]	84	[− 12.0; − 1.7]	●○○○
WOMAC-P	12 mo	3	130	− 5.9 [− 9.6; − 2.2]	95	[− 21.9; 10.1]	●○○○
KOOS-P	3 mo	6	517	+ 20.2 [16.4; 24.0]	53	[10.4; 30.0]	●●○○
KOOS-P	6 mo	8	590	+ 23.6 [21.6; 25.7]	0.1	[21.1; 26.1]	●●○○
KOOS-P	12 mo	4	366	+ 24.8 [20.6; 28.9]	35	[14.2; 35.3]	●●○○

WMD weighted mean difference; CI confidence interval; WOMAC-T WOMAC-Total; WOMAC-P WOMAC-Pain; KOOS-P KOOS-Pain. Negative WMD for VAS/WOMAC = improvement. Positive WMD for KOOS = improvement

**Fig. 3:**
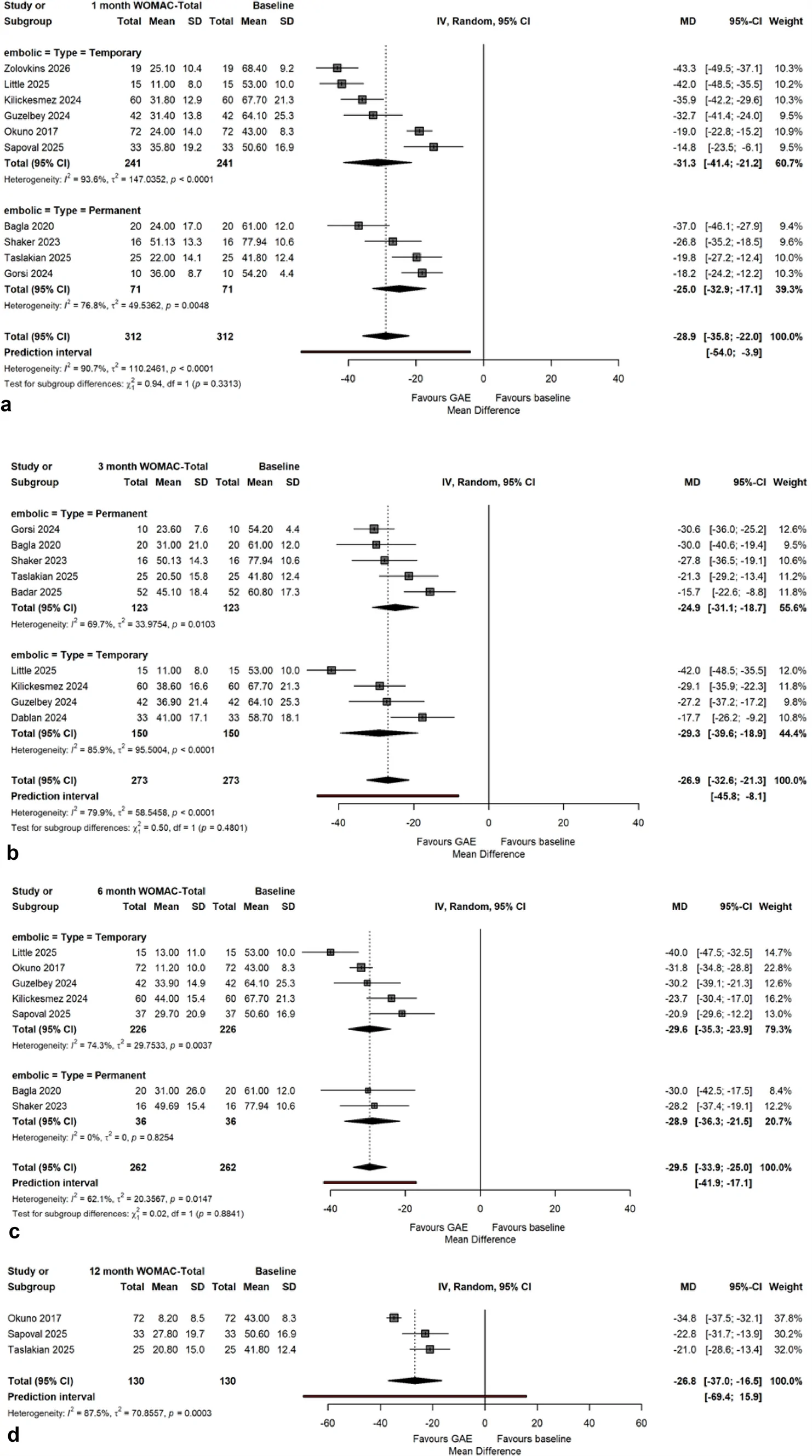
Forest plots of pooled WOMAC-Total. Forest plots of pooled WOMAC-Total change from baseline after genicular artery embolization at **a** 1 month (k = 10; WMD = −  28.9; I^2^ = 91%), **b** 3 months (k = 9; WMD = −  26.9; I^2^ = 80%), **c** 6 months (k = 7; WMD = −  29.5; I^2^ = 62%), and **d** 12 months (k = 3; WMD = −  26.8; I^2^ = 88%)

**Fig. 4:**
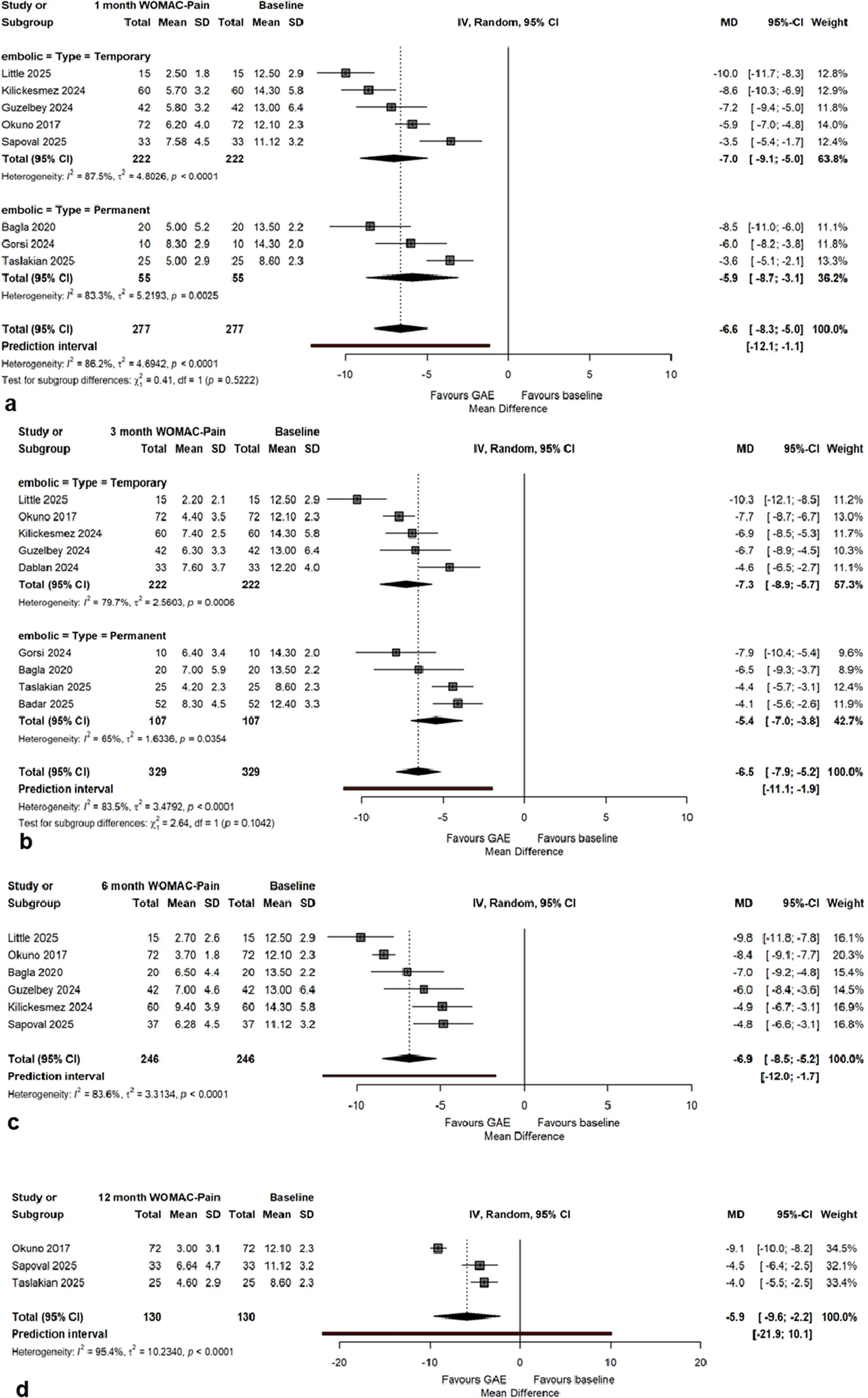
Forest plots of pooled WOMAC-Pain. Forest plots of pooled WOMAC-Pain (0–20) change from baseline after genicular artery embolization at **a** 1 month (k = 8; WMD = −  6.6; I^2^ = 86%), **b** 3 months (k = 9; WMD = − 6.5; I^2^ = 84%), **c** 6 months (k = 6; WMD = −  6.9; I^2^ = 84%), and **d** 12 months (k = 3; WMD = −  5.9; I^2^ = 95%), with subgroup analysis by embolic material type (permanent vs. temporary)

WOMAC-Total improved by − 28.9 at 1 month (51% reduction; k = 10; I^2^ = 91%), − 26.9 at 3 months (44% reduction; k = 9; I^2^ = 80%), − 29.5 at 6 months (51% reduction; k = 7; I^2^ = 62%), and − 26.8 at 12 months (59% reduction; k = 3; I^2^ = 88%), (Table 2; Fig. 3). All WOMAC-Total reductions exceeded the MCID (reduction ≥ 16 points) [26]; however, the 12-month prediction interval (− 69.4 to 15.9) crossed zero. WOMAC-Pain showed consistent reductions of − 5.9 to − 6.9 across all timepoints (k = 3–9; Fig. 4).

**Fig. 5:**
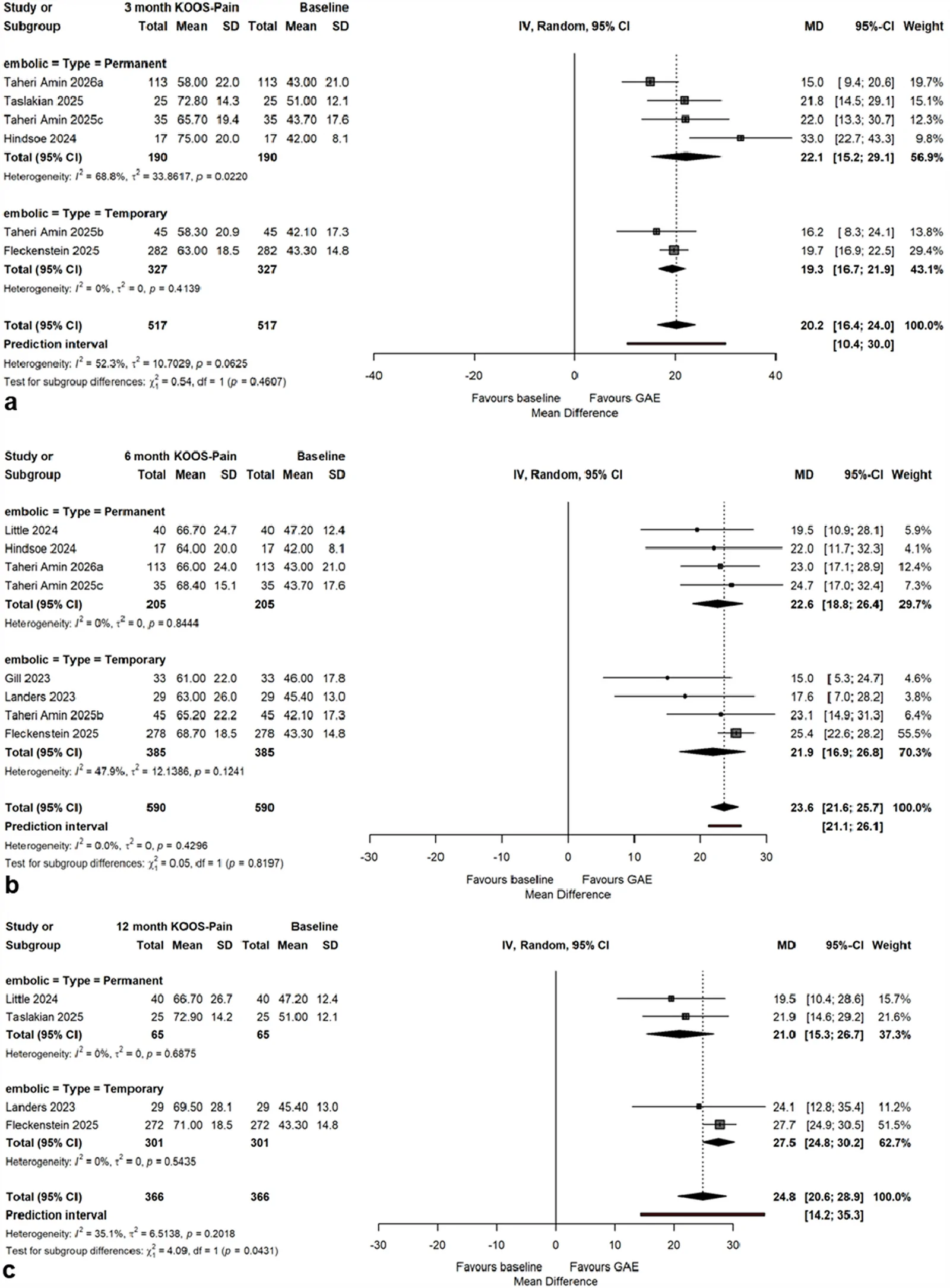
Forest plots of pooled KOOS-Pain. Forest plots of pooled KOOS-Pain change from baseline after genicular artery embolization at **a** 3 months (k = 6; WMD =  + 20.2; I^2^ = 53%), **b** 6 months (k = 8; WMD =  + 23.6; I^2^ = 0.1%), and **c** 12 months (k = 4; WMD =  + 24.8; I^2^ = 35%). The 6-month analysis demonstrated the lowest heterogeneity of any outcome (I^2^ = 0.1%), indicating consistent improvement across all included studies

KOOS-Pain improved by + 20.2 at 3 months (46% improvement; k = 6; I^2^ = 53%), + 23.6 at 6 months (54% improvement; k = 8; I^2^ = 0.1%), and + 24.8 at 12 months (56% improvement; k = 4; I^2^ = 35%), all exceeding the MCID (improvement ≥ 10 points) [24] (Table 2; Fig. 5). The 6-month analysis demonstrated the lowest heterogeneity of any outcome in this review.

### Safety Outcomes

**Fig. 6:**
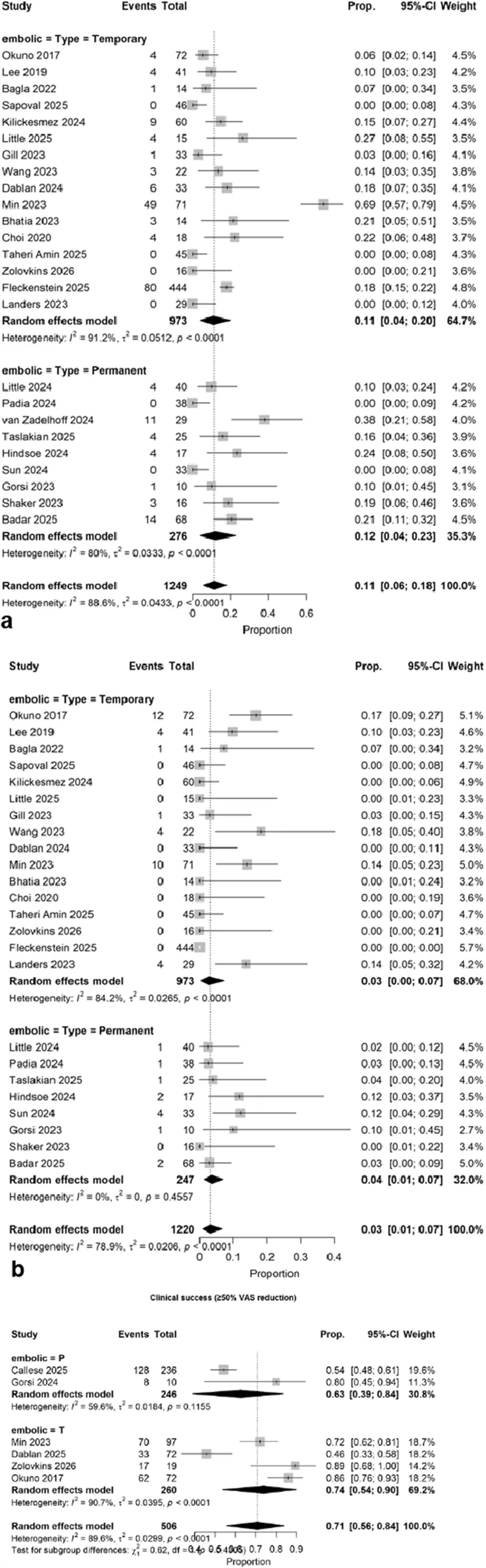
Pooled adverse events. Pooled adverse events proportion forest plots: **a** transient skin discoloration (k = 25; 11%; 95% CI 6–18%; I^2^ = 89%); **b** subcutaneous hematoma (k = 24; 3%; 95% CI 1–7%; I^2^ = 79%); **c** clinical success defined as ≥ 50% VAS reduction (k = 6; 71%; 95% CI 56–84%). No major adverse events were reported

The most common adverse events were transient skin discoloration (pooled 11%; 95% CI 6–18%; I^2^ = 89%) and subcutaneous hematoma (3%; 95% CI 1–7%; I^2^ = 79%). No complications of modified CIRSE grade 3 or higher were reported in any included study [64]. Clinical success (≥ 50% VAS reduction) was achieved in 71% of patients (95% CI 56–84%; k = 6). Pooled adverse event proportions are reported in Table 3. Forest plots for skin discoloration, hematoma, and clinical success are shown in Fig. 6. Total-knee arthroplasty conversion and technical success plots are in Supplementary Fig. S1.

**Table 3:** Pooled adverse event proportions (Freeman–Tukey double arcsine transformation)

Adverse event	k	Events/Total	Pooled proportion [95% CI]	I^2^ (%)
Transient skin discoloration	25	209/1,249	11% [6–18%]	89
Subcutaneous hematoma	24	48/1,220	3% [1–7%]	79

Only adverse events amenable to quantitative pooling are shown. Self-limiting groin hematomas and transient paresthesia were reported in individual studies but could not be pooled due to inconsistent reporting

### Subgroup and Sensitivity Analyses

No significant subgroup interaction between permanent and temporary embolic agents were found for any outcome at any timepoint (all subgroup interaction *p* > 0.05; Table 2). Leave-one-out analysis confirmed the stability of pooled estimates. Sequential exclusion of studies from probable overlapping cohorts [15, 36–39, 41, 42] did not materially alter the results (range of change: 0.3–1.8 points). Funnel plot inspection for VAS 1-month (k = 13) showed mild asymmetry (Supplementary Figs. S2–S3).

### Certainty of Evidence

The certainty of evidence was assessed using the GRADE framework (Supplementary Tables S8–S10). Starting certainty was low given the predominance of single-arm studies at serious risk of bias. After applying downgrades for inconsistency and imprecision, final certainty ranged from very low to low for all outcomes. KOOS-Pain at 6 months achieved low certainty (I^2^ ≈ 0%). Adverse event estimates were rated very low (Table 2).

## Discussion

In this systematic review and meta-analysis of 45 studies and 2,205 patients, GAE produced VAS reductions exceeding the 20-point MCID at all assessed timepoints, from − 37.5 (55% reduction; 95% CI − 43.3 to − 31.8) at 1 month to − 37.1 (54% reduction; 95% CI − 46.0 to − 28.3) at 12 months [46]. WOMAC-Total decreased by 27–29 points (44–59% reduction), sustained through 12 months. KOOS-Pain improved by 20–25 points (46–56% improvement), with the 6-month estimate reaching I^2^ = 0.1% across eight studies. All VAS and KOOS-Pain prediction intervals excluded zero through 12 months, indicating that clinically meaningful reductions can be expected even in new clinical trials.

VAS pooled estimates were consistent with those reported in prior meta-analyses (− 37.5 vs. − 38.5 at 1 month and − 37.1 vs. − 40.5 at 12 months in Chlorogiannis et al. [13]), confirming stability across an expanded evidence base. WOMAC-Total estimates were 6–9 points higher (− 29.0 vs. − 21.1 at 1 month), likely reflecting the contribution of the Fleckenstein cohort (*n* = 333) [17]. KOOS-Pain at 6 months reached I^2^ = 0.1%, in contrast with the I^2^ values of 57–93% reported across all outcomes by Chlorogiannis et al. [13]. The availability of 6–8 studies including the KOOS as an outcome parameter, compared with the 5 previously pooled, may account for this improvement in consistency.

While pooled pre-post estimates consistently exceeded MCID thresholds, the two adequately blinded sham-controlled RCTs available for this review both reported negative primary outcomes [14, 15]. Van Zadelhoff et al. found a between-group difference of only 3.0 points in KOOS-Pain at 4 months (95% CI − 7.1 to 13.0) [14], and Landers et al. reported no statistically significant difference in any KOOS subscale at 12 months [15]. A subsequent 12-month follow-up of the van Zadelhoff trial, published after the cutoff date of our systematic search and therefore not included in the quantitative synthesis, confirmed sustained equivalent pain reduction in both GAE and sham arms, with no significant between-group differences in pain, function, or synovitis, consistent with a long-term placebo response rather than a specific treatment effect [65]. Methodological aspects of this trial, particularly its time frames and confidence interval framework, have prompted ongoing scientific discussion.

This discrepancy likely reflects mechanisms inherent to uncontrolled designs. While GAE has a plausible biological rationale [5], the procedure also involves arterial access, conscious sedation, and structured post-procedural follow-up, all of which amplify expectancy effects and elicit placebo responses of clinically meaningful magnitude. Because patients are enrolled at peaks of symptomatic severity, regression toward their individual baseline tends to produce improvement irrespective of intervention. The natural symptomatic fluctuation of knee osteoarthritis introduces further within-patient variability that single-arm designs cannot adjust for. Whether the observed effect reflects mechanistic efficacy or perceived benefit therefore remains uncertain until adequately powered sham-controlled trials are available. The GRADE assessment (very low to low certainty) reflects this fundamental limitation of the evidence base.

No significant subgroup interaction between permanent and temporary embolic agents was observed at any timepoint (all interaction *p* > 0.05). This finding is consistent with the subgroup analysis by Chlorogiannis et al. [13]. However, these indirect, between-study comparisons are underpowered and vulnerable to substantial confounding from differences in patient selection, embolization technique, and follow-up duration. Absence of a significant subgroup interaction does not establish equivalence between embolic agents. This subgroup analysis should therefore be interpreted as hypothesis-generating only, and any conclusion regarding non-inferiority or equivalence would require dedicated head-to-head randomized trials. The systematic identification of overlapping cohorts prevented double-counting of shared patients across at least seven institutional cohorts. As publications from high-volume centers increase, future reviews should adopt comparable frameworks.

This study had several limitations. Heterogeneity was considerable (I^2^ > 80%) for most VAS and WOMAC outcomes, reflecting variation in patient selection, embolic materials, technique, and outcome assessment. This, combined with the observational design of 42 of 45 studies, yielded GRADE certainty of very low to low. Only three RCTs were available, all sham-controlled. However, only two maintained adequate blinding beyond 1 month, as the Bagla 2022 sham group crossed over completely at 1 month [16]. The overlap analysis relied partly on circumstantial evidence; therefore undetected overlap may persist. Values extracted from figures using WebPlotDigitizer carry greater uncertainty than reported values, although sensitivity analysis excluding these did not alter conclusions. Pooled adverse event proportions likely underestimate true incidence given the observational design of most included studies, the absence of standardized adverse events classification frameworks across cohorts, and likely publication bias toward favorable safety profiles. At 12 months, WOMAC-Total and WOMAC-Pain prediction intervals crossed zero, constraining generalizability.

The current evidence base is mainly dominated by single-arm studies at serious risk of bias, and the limited RCT evidence (3 trials, 138 patients) has yet to demonstrate superiority over sham. Several ongoing trials are positioned to address these gaps directly. The GAUCHO trial [66], is a randomized study comparing clinical efficacy of imipenem/cilastatin versus permanent microspheres up to 12-months. The GRAVITY trial [67] is a randomized controlled study comparing GAE versus observation in 100 patients, with ≥ 50% WOMAC reduction at 6 months as the primary endpoint and MRI-based mechanistic endpoints, currently recruiting. The GENESIS II trial [68], a double-blind sham-controlled RCT targeting 110 patients with 24-month follow-up (primary completion 2027), directly addresses the limitations of prior sham-controlled studies through standardized KOOS primary endpoints and MRI-based synovial assessment. Together, these trials represent the most rigorous prospective evaluation of GAE to date and are expected to determine whether the consistent treatment signal observed across 45 uncontrolled studies reflects a true biological effect.

In conclusion, GAE produced large within-group pain reductions and functional improvements across VAS, WOMAC, and KOOS exceeding MCID thresholds through 12 months, but these reflect an observational signal from predominantly single-arm studies. The two adequately blinded sham-controlled RCTs did not demonstrate between-group superiority over sham, GRADE certainty of evidence was very low to low, and no difference between embolic agents was observed. There is no high-quality evidence demonstrating superiority over sham. Adequately powered sham-controlled trials currently in progress are needed to confirm or refute this signal.

## Supplementary Information

Below is the link to the electronic supplementary material.

Supplementary file1 (PNG 84 KB)

Supplementary file2 (PNG 85 KB)

Supplementary file3 (PNG 141 KB)

Supplementary file4 (PNG 153 KB)

Supplementary file5 (PNG 90 KB)

Supplementary file6 (DOCX 18 KB)

Supplementary file7 (DOCX 15 KB)

Supplementary file8 (DOCX 14 KB)

Supplementary file9 (DOCX 16 KB)

Supplementary file10 (DOCX 16 KB)

Supplementary file11 (DOCX 21 KB)

Supplementary file12 (DOCX 15 KB)

Supplementary file13 (DOCX 17 KB)

Supplementary file14 (DOCX 16 KB)

Supplementary file15 (DOCX 17 KB)

Supplementary file16 (DOCX 18 KB)
