# Supplementary material for: Outcome and Safety of Genicular Artery Embolization for Knee Osteoarthritis: A Systematic Review and Meta-Analysis
Source: Cardiovasc Intervent Radiol. 2026 Jun 16;49(9):1682–97. doi: 10.1007/s00270-026-04502-7 (PMC13597475; doi:10.1007/s00270-026-04502-7)
Supplement: Supplementary file 1 [file 270_2026_4502_MOESM10_ESM.docx]

| **Study** | **Comparison** | **n** | **D1 Confound** | **D2 Select** | **D3 Classif** | **D4 Deviat** | **D5 Missing** | **D6 Measure** | **D7 Report** | **Overall** |
| --- | --- | --- | --- | --- | --- | --- | --- | --- | --- | --- |
| **Guzelbey 2024a** | Lipiodol vs IPM-CS | 42 | **Serious** | Moderate | Low | Low | Low | Moderate | Low | **Serious** |
| **Guzelbey 2024b** | Micro vs Macrocatheter | 79 | **Serious** | Moderate | Low | Low | Low | Moderate | Low | **Serious** |
| **Kilickesmez 2024** | TPA vs TFA | 60 | **Serious** | Moderate | Low | Low | Low | Moderate | Low | **Serious** |
| **Bhatia 2023** | ES vs IPM/CS | 14 | **Serious** | **Serious** | Low | Low | **Serious** | Moderate | Low | **Critical** |
| **Lee 2019** | Mild-mod vs Severe OA | 41 | **Serious** | Moderate | Low | Moderate | Moderate | Moderate | Low | **Serious** |
| **Badar 2025c** | Perm vs Temp agents | 68 | **Serious** | **Serious** | Low | Low | Moderate | Moderate | Low | **Serious** |
| **Cusumano 2024** | 24-mo FU extension | 40 | Moderate | Low | Low | Low | Moderate | Moderate | Low | Moderate |
