# Supplementary material for: Outcome and Safety of Genicular Artery Embolization for Knee Osteoarthritis: A Systematic Review and Meta-Analysis
Source: Cardiovasc Intervent Radiol. 2026 Jun 16;49(9):1682–97. doi: 10.1007/s00270-026-04502-7 (PMC13597475; doi:10.1007/s00270-026-04502-7)
Supplement: Supplementary file 2 [file 270_2026_4502_MOESM11_ESM.docx]

| **Study** | **Design** | **n** | **D1 Confound** | **D2 Select** | **D3 Classif** | **D4 Deviat** | **D5 Missing** | **D6 Measure** | **D7 Report** | **Overall** |
| --- | --- | --- | --- | --- | --- | --- | --- | --- | --- | --- |
| **Okuno 2017** | Prosp | 72 | **Critical** | Moderate | Low | Low | Moderate | Moderate | Low | **Serious** |
| **Landers 2020** | Prosp pilot | 10 | **Critical** | Moderate | Low | Moderate | Moderate | Moderate | Low | **Serious** |
| **Choi 2020** | Retro | 18 | **Critical** | Moderate | Low | Low | Low | Moderate | Low | **Serious** |
| **Bagla 2020** | Prosp pilot | 20 | **Critical** | Low | Low | Low | Low | Moderate | Low | **Serious** |
| **Padia 2021** | Prosp IDE | 40 | **Critical** | Low | Low | Low | Low | Moderate | Low | **Serious** |
| **van Zadelhoff 2021** | Retro | 54 | **Critical** | Moderate | Low | Low | Moderate | Moderate | Low | **Serious** |
| **Russu 2022** | Prosp series | 17 | **Critical** | Moderate | Low | Low | Low | **Serious** | Low | **Serious** |
| **Sapoval 2023** | Prosp FIH | 22 | **Critical** | Low | Low | Low | Low | Moderate | Low | **Serious** |
| **Gill 2023** | Prosp | 33 | **Critical** | Moderate | Low | Low | Low | Moderate | Low | **Serious** |
| **Min 2023** | Retro | 71 | **Critical** | Moderate | Low | Low | Low | Moderate | Moderate | **Serious** |
| **Wang 2023** | Prosp pilot | 22 | **Critical** | Moderate | Low | Low | Low | Moderate | Low | **Serious** |
| **Taslakian 2023** | Prosp interim | 16 | **Critical** | Moderate | Low | Low | Moderate | Moderate | Low | **Serious** |
| **Shaker 2023** | Prosp | 16 | **Critical** | Moderate | Low | Low | Low | Moderate | Low | **Serious** |
| **Hindso 2024a** | Prosp imaging | 17 | **Critical** | Low | Low | Low | Low | Moderate | Low | **Serious** |
| **Hindso 2024b** | Prosp | 17 | **Critical** | Low | Low | Low | Low | Moderate | Low | **Serious** |
| **Little 2024** | Prosp NIHR | 46 | **Critical** | Low | Low | Low | Moderate | Moderate | Low | **Serious** |
| **Sun 2024** | Prosp | 33 | **Critical** | Moderate | Low | Low | Low | Moderate | Low | **Serious** |
| **Dablan 2024** | Retro | 33 | **Critical** | Moderate | Low | Low | Low | Moderate | Low | **Serious** |
| **Gorsi 2024** | Prosp | 10 | **Critical** | Moderate | Low | Low | Low | Moderate | Low | **Serious** |
| **Fleckenstein 2025** | Retro (DB) | 333 | **Critical** | Low | Low | Low | Moderate | Moderate | Low | **Serious** |
| **Taheri Amin 2025a** | Prosp | 43 | **Critical** | Moderate | Low | Low | Moderate | **Serious** | Low | **Serious** |
| **Taheri Amin 2025b** | Prosp explor | 35 | **Critical** | Moderate | Low | Low | Low | Moderate | Low | **Serious** |
| **Taheri Amin 2025c** | Prosp RGM | 45 | **Critical** | Moderate | Low | Low | Low | Moderate | Low | **Serious** |
| **Callese 2025** | Retro | 236 | **Critical** | Low | Low | Low | Low | Moderate | Low | **Serious** |
| **Sapoval 2025** | Prosp | 46 | **Critical** | Low | Low | Low | Moderate | Moderate | Low | **Serious** |
| **Taslakian 2025** | Prosp IDE | 25 | **Critical** | Low | Low | Low | Moderate | Moderate | Low | **Serious** |
| **Badar 2025a** | Retro | 34 | **Critical** | Moderate | Low | Low | Moderate | Moderate | Low | **Serious** |
| **Badar 2025b** | Retro MRI | 39 | **Critical** | Moderate | Low | Low | Low | Moderate | Low | **Serious** |
| **Little 2025** | Prosp FIH | 15 | **Critical** | Moderate | Low | Low | Low | Moderate | Low | **Serious** |
| **Dablan 2025** | Retro ML | 66 | **Critical** | Moderate | Low | Low | Moderate | Moderate | Low | **Serious** |
| **Harrison 2025** | Prosp neuro | 30 | **Critical** | Low | Low | Low | Low | Moderate | Low | **Serious** |
| **Taheri Amin 2026a** | Prosp | 113 | **Critical** | Low | Low | Low | Low | Moderate | Low | **Serious** |
| **Taheri Amin 2026b** | Prosp reGAE | 55 | **Critical** | Moderate | Low | Low | Low | Moderate | Low | **Serious** |
| **Mabud 2026** | Retro explor | 25 | **Critical** | Low | Low | Low | Moderate | Moderate | Low | **Serious** |
| **Zolovkins 2026** | Prosp | 16 | **Critical** | Moderate | Low | Low | Low | Moderate | Low | **Serious** |
