# Supplementary material for: Outcome and Safety of Genicular Artery Embolization for Knee Osteoarthritis: A Systematic Review and Meta-Analysis
Source: Cardiovasc Intervent Radiol. 2026 Jun 16;49(9):1682–97. doi: 10.1007/s00270-026-04502-7 (PMC13597475; doi:10.1007/s00270-026-04502-7)
Supplement: Supplementary file 3 [file 270_2026_4502_MOESM12_ESM.docx]

| **Domain** | **Judgment distribution** | **Support for judgment** |
| --- | --- | --- |
| **D1. Confounding** | Critical (35/35) | All single-arm without control group. Cannot distinguish treatment effect from placebo response, natural disease course, regression to the mean, or concurrent conservative therapies (NSAIDs, PT, injections allowed in most studies). |
| **D2. Selection** | Low (15) / Moderate (20) | Low: prospective studies with clear eligibility and consecutive enrollment (Padia, Bagla 2020, Callese, Fleckenstein, Little 2024, Harrison, Sapoval 2023/2025, Hindso, Taslakian 2025, Taheri Amin 2026a). Moderate: retrospective designs with potential selection bias or post-hoc exclusions. |
| **D3. Classification** | Low (35/35) | GAE well-defined in all studies: target arteries, embolic material, and angiographic endpoint clearly described. No ambiguity in intervention classification. |
| **D4. Deviations** | Low (33) / Moderate (2) | Most patients received the planned intervention. Moderate: Landers 2020 (2 different embolic agents used in same study); Taheri Amin 2025a (non-validated questionnaire may alter patient perception). |
| **D5. Missing data** | Low (24) / Moderate (11) | Low: studies with 100% follow-up or minimal attrition (<15%). Moderate: Fleckenstein 2025 (82% at 12mo), Sapoval 2025 (74% knees at 12mo), Taslakian 2023 (37.5% at 12mo), Taslakian/Mabud 2025/2026 (84% at 12mo), van Zadelhoff 2021 (12/66 excluded for MRI quality), Badar 2025a (44% at 2yr). |
| **D6. Measurement** | Moderate (32) / Serious (3) | Moderate: self-reported PROMs (VAS, WOMAC, KOOS) without blinding; expectation bias inherent in open-label designs. Serious: Russu 2022 (reversed WOMAC 0–100 scale where higher = better, inconsistent with standard Likert); Taheri Amin 2025a (non-validated 10-item NRS questionnaire). |
| **D7. Reporting** | Low (30) / Moderate (5) | Low: pre-registered studies with outcomes aligned to protocol. Moderate: unregistered retrospective studies reporting multiple timepoints and post-hoc subanalyses without clear a priori specification (Min 2023, Dablan 2025). |
