# Supplementary material for: Outcome and Safety of Genicular Artery Embolization for Knee Osteoarthritis: A Systematic Review and Meta-Analysis
Source: Cardiovasc Intervent Radiol. 2026 Jun 16;49(9):1682–97. doi: 10.1007/s00270-026-04502-7 (PMC13597475; doi:10.1007/s00270-026-04502-7)
Supplement: Supplementary file 4 [file 270_2026_4502_MOESM13_ESM.docx]

| **Group** | **Classification** | **Studies involved** | **Key evidence** | **Decision** |
| --- | --- | --- | --- | --- |
| **CONFIRMED OVERLAPS** |  |  |  |  |
| UCLA | Confirmed | Padia 2021 (n=40), Cusumano/Padia 2024 (n=40), Callese 2025 (n=236) | Padia 2024: "same study cohort previously published." Callese: "some study subjects have been previously reported." Padia 40 pts ⊂ Callese 236. | Retain Callese 2025 (largest, most recent). Exclude Padia 2021 and Cusumano 2024 from primary analysis. |
| NYU Langone | Confirmed | Taslakian 2023 (n=16), Taslakian 2025 (n=25), Mabud 2026 (n=25) | Taslakian 2025: "all patients in previous short-term study included." Mabud: "data from NCT04379700 [Taslakian 2025]." | Retain Taslakian 2025 (final analysis). Exclude Taslakian 2023 and Mabud 2026. |
| GENESIS, UK | Confirmed | Little 2024 (n=46), Harrison 2025 (n=30) | Harrison: "forms part of the GENESIS study." Same IRAS registration. Harrison 30 ⊂ Little 46. | Retain Little 2024 (complete cohort). Exclude Harrison 2025. |
| Edogawa, Tokyo | Confirmed | Okuno 2017 (n=72), van Zadelhoff 2021 (n=54) | van Zadelhoff: "24 patients from this cohort were included." 24/54 from Okuno. | Retain both (partial overlap, 33%). Sensitivity analysis excluding van Zadelhoff. |
| Paris (HEGP) | Confirmed | Sapoval 2023 (n=22), Sapoval 2025 (n=46) | Sapoval 2025: "22 of these 50 GAEs previously published." Sapoval 2023 ⊂ Sapoval 2025. | Retain Sapoval 2025 (complete). Exclude Sapoval 2023 at shared timepoints. |
| Copenhagen | Confirmed | Hindso 2024 clinical (n=17), Hindso 2024 imaging (n=17) | Imaging paper: "expands our previous prospective research." Same NCT05360329. | Retain clinical paper. Imaging paper reports different outcomes (no double-counting). |
| U Chicago | Confirmed | Badar 2025a (n=34), Badar 2025b (n=39), Badar 2025c (n=68) | Badar 2025c: "44 patients previously reported in [refs = 2025a, 2025b]." All ⊂ 2025c. | Retain Badar 2025c (largest). Exclude 2025a and 2025b. |
| **PROBABLE OVERLAPS** |  |  |  |  |
| Geelong | Probable | Landers 2020 (n=10), Landers 2023 RCT (n=59), Gill 2023 (n=33) | Same center, same IR (S.L.). RCT references pilot. Different trial registrations. No explicit overlap statement. | Retain all independently. Flag in sensitivity analysis. |
| Istanbul | Probable | Guzelbey 2024a (n=79), Guzelbey 2024b (n=42), Dablan 2024 (n=33), Kilickesmez 2024 (n=60), Dablan 2025 (n=66) | Single center, single IR, heavily overlapping periods. No cross-referencing between papers. | Include all (each applies different inclusion criteria); note in limitations. |
