# Supplementary material for: Outcome and Safety of Genicular Artery Embolization for Knee Osteoarthritis: A Systematic Review and Meta-Analysis
Source: Cardiovasc Intervent Radiol. 2026 Jun 16;49(9):1682–97. doi: 10.1007/s00270-026-04502-7 (PMC13597475; doi:10.1007/s00270-026-04502-7)
Supplement: Supplementary file 5 [file 270_2026_4502_MOESM14_ESM.docx]

| **Study** | **Institution** | **Enrollment period** |
| --- | --- | --- |
| Bagla 2022 (RCT) | VIP-NOVA / UNC | Jun 2018 – May 2019 |
| van Zadelhoff 2024 (RCT) | Erasmus MC, Rotterdam | Jun 2019 – Dec 2021 |
| Fleckenstein 2025 | Charité, Berlin | Dec 2022 – Jul 2024 |
| Bagla 2020 | VIV / UNC | Jan 2017 – Jan 2018 |
| Little 2025 | Tashkent, Uzbekistan | Dec 2023 – Feb 2024 |
| Sun 2024 | Ningxia, China | Oct 2020 – Jan 2022 |
| Wang 2023 | NCKU Hospital, Taiwan | Jan 2020 – Jan 2022 |
| Gorsi 2024 | PGIMER, Chandigarh | Feb 2021 – Mar 2022 |
| Min 2023 | Konkuk U, Seoul | Aug 2019 – Jan 2022 |
| Bhatia 2023 | Okuno Clinic, Tokyo | Mar 3–27, 2018 |
| Choi 2020 | SNUH, Seoul | Apr – Oct 2019 |
| Zolovkins 2026 | Sant'Andrea, Rome | Apr – Jul 2024 |
| Lee 2019 | H Plus Yangji, Seoul | Jun 2017 – Jul 2018 |
| Russu 2022 | Monza Hospital, Bucharest | Not stated |
| Shaker 2023 | Ain Shams U, Cairo | Not stated |
| Taheri Amin 2025a | U Hospital Düsseldorf | Not stated |
| Taheri Amin 2025b | U Hospital Düsseldorf | Jan – Dec 2024 |
| Taheri Amin 2025c | U Hospital Düsseldorf | Dec 2024 – Apr 2025 |
| Taheri Amin 2026a | U Hospital Düsseldorf | Jan 2023 – Dec 2024 |
| Taheri Amin 2026b | U Hospital Düsseldorf | Jan 2024 – Jun 2025 |
