# Supplementary material for: Outcome and Safety of Genicular Artery Embolization for Knee Osteoarthritis: A Systematic Review and Meta-Analysis
Source: Cardiovasc Intervent Radiol. 2026 Jun 16;49(9):1682–97. doi: 10.1007/s00270-026-04502-7 (PMC13597475; doi:10.1007/s00270-026-04502-7)
Supplement: Supplementary file 6 [file 270_2026_4502_MOESM15_ESM.docx]

| **Outcome** | **Time** | **k** | **N** | **Risk of bias** | **Incon- sistency** | **Indirect- ness** | **Impre- cision** | **Pub. bias** | **Large effect** | **Certainty** |
| --- | --- | --- | --- | --- | --- | --- | --- | --- | --- | --- |
| **VAS** | 1 mo | 13 | 440 | **▼** | **▼** | **—** | **—** | **▼** | **▲** | **⬤○○○ Very low** |
| **VAS** | 3 mo | 12 | 653 | **▼** | **▼** | **—** | **—** | **—** | **▲** | **⬤○○○ Very low** |
| **VAS** | 6 mo | 11 | 683 | **▼** | **▼** | **—** | **—** | **—** | **▲** | **⬤○○○ Very low** |
| **VAS** | 12 mo | 7 | 495 | **▼** | **▼▼** | **—** | **—** | **—** | **▲** | **⬤○○○ Very low** |
| **WOMAC-T** | 1 mo | 10 | 312 | **▼** | **▼** | **—** | **—** | **—** | **▲** | **⬤○○○ Very low** |
| **WOMAC-T** | 3 mo | 9 | 273 | **▼** | **▼** | **—** | **—** | **—** | **▲** | **⬤○○○ Very low** |
| **WOMAC-T** | 6 mo | 7 | 262 | **▼** | **—** | **—** | **—** | **—** | **▲** | **⬤⬤○○ Low** |
| **WOMAC-T** | 12 mo | 3 | 130 | **▼** | **▼▼** | **—** | **▼** | **—** | **▲** | **⬤○○○ Very low** |
| **WOMAC-P** | 1 mo | 8 | 277 | **▼** | **▼** | **—** | **—** | **—** | **—** | **⬤○○○ Very low** |
| **WOMAC-P** | 3 mo | 9 | 329 | **▼** | **▼** | **—** | **—** | **—** | **—** | **⬤○○○ Very low** |
| **WOMAC-P** | 6 mo | 6 | 246 | **▼** | **▼** | **—** | **—** | **—** | **—** | **⬤○○○ Very low** |
| **WOMAC-P** | 12 mo | 3 | 130 | **▼** | **▼▼** | **—** | **▼** | **—** | **—** | **⬤○○○ Very low** |
| **KOOS-P** | 3 mo | 6 | 517 | **▼** | **—** | **—** | **—** | **—** | **▲** | **⬤⬤○○ Low** |
| **KOOS-P** | 6 mo | 8 | 590 | **▼** | **—** | **—** | **—** | **—** | **▲** | **⬤⬤○○ Low** |
| **KOOS-P** | 12 mo | 4 | 366 | **▼** | **—** | **—** | **—** | **—** | **▲** | **⬤⬤○○ Low** |
| **Skin discolor.** | — | 25 | 1,249 | **▼** | **▼** | **—** | **—** | **—** | **—** | **⬤○○○ Very low** |
| **Hematoma** | — | 24 | 1,220 | **▼** | **▼** | **—** | **—** | **—** | **—** | **⬤○○○ Very low** |
