# Supplementary material for: Outcome and Safety of Genicular Artery Embolization for Knee Osteoarthritis: A Systematic Review and Meta-Analysis
Source: Cardiovasc Intervent Radiol. 2026 Jun 16;49(9):1682–97. doi: 10.1007/s00270-026-04502-7 (PMC13597475; doi:10.1007/s00270-026-04502-7)
Supplement: Supplementary file 7 [file 270_2026_4502_MOESM16_ESM.docx]

| **Outcome** | **Timepoint** | **No. of studies (k)** | **No. of patients** | **Effect WMD [95% CI]** | **I² (%)** | **Prediction interval** | **Certainty of evidence** | **Footnotes** |
| --- | --- | --- | --- | --- | --- | --- | --- | --- |
| **VAS** | 1 mo | 13 | 440 | −37.5 [−43.3; −31.8] | 86% | [−59.4; −15.6] | **⬤○○○ Very low** | a,b,c,d |
| **VAS** | 3 mo | 12 | 653 | −34.5 [−39.5; −29.4] | 84% | [−52.7; −16.3] | **⬤○○○ Very low** | a,b,d |
| **VAS** | 6 mo | 11 | 683 | −36.7 [−42.2; −31.2] | 86% | [−56.2; −17.2] | **⬤○○○ Very low** | a,b,d |
| **VAS** | 12 mo | 7 | 495 | −37.1 [−46.0; −28.3] | 90% | [−66.2; −8.1] | **⬤○○○ Very low** | a,e,d |
| **WOMAC-T** | 1 mo | 10 | 312 | −28.9 [−35.8; −22.0] | 91% | [−54.0; −3.9] | **⬤○○○ Very low** | a,b,d |
| **WOMAC-T** | 3 mo | 9 | 273 | −26.9 [−32.6; −21.3] | 80% | [−45.8; −8.1] | **⬤○○○ Very low** | a,b,d |
| **WOMAC-T** | 6 mo | 7 | 262 | −29.5 [−33.9; −25.0] | 62% | [−41.9; −17.1] | **⬤⬤○○ Low** | a,d |
| **WOMAC-T** | 12 mo | 3 | 130 | −26.8 [−37.0; −16.5] | 88% | [−69.4; 15.9] | **⬤○○○ Very low** | a,e,f,d |
| **WOMAC-P** | 1 mo | 8 | 277 | −6.6 [−8.3; −5.0] | 86% | [−12.1; −1.1] | **⬤○○○ Very low** | a,b |
| **WOMAC-P** | 3 mo | 9 | 329 | −6.5 [−7.9; −5.2] | 84% | [−11.1; −1.9] | **⬤○○○ Very low** | a,b |
| **WOMAC-P** | 6 mo | 6 | 246 | −6.9 [−8.5; −5.2] | 84% | [−12.0; −1.7] | **⬤○○○ Very low** | a,b |
| **WOMAC-P** | 12 mo | 3 | 130 | −5.9 [−9.6; −2.2] | 95% | [−21.9; 10.1] | **⬤○○○ Very low** | a,e,f |
| **KOOS-P** | 3 mo | 6 | 517 | +20.2 [16.4; 24.0] | 53% | [10.4; 30.0] | **⬤⬤○○ Low** | a,d |
| **KOOS-P** | 6 mo | 8 | 590 | +23.6 [21.6; 25.7] | 0.1% | [21.1; 26.1] | **⬤⬤○○ Low** | a,g,d |
| **KOOS-P** | 12 mo | 4 | 366 | +24.8 [20.6; 28.9] | 35% | [14.2; 35.3] | **⬤⬤○○ Low** | a,d |
| **Skin discolor.** | — | 25 | 1,249 | 11% [6–18%] | 89% | — | **⬤○○○ Very low** | a,h |
| **Hematoma** | — | 24 | 1,220 | 3% [1–7%] | 79% | — | **⬤○○○ Very low** | a,h |
