# Supplementary material for: Outcome and Safety of Genicular Artery Embolization for Knee Osteoarthritis: A Systematic Review and Meta-Analysis
Source: Cardiovasc Intervent Radiol. 2026 Jun 16;49(9):1682–97. doi: 10.1007/s00270-026-04502-7 (PMC13597475; doi:10.1007/s00270-026-04502-7)
Supplement: Supplementary file 8 [file 270_2026_4502_MOESM6_ESM.docx]

| **Database** | **Line** | **Search Terms** |
| --- | --- | --- |
| **PubMed (MEDLINE)** |  |  |
|  | #1 | (genicular[tiab] OR geniculate[tiab] OR "genicular artery"[tiab] OR "genicular arteries"[tiab] OR "descending genicular"[tiab] OR "superior medial genicular"[tiab] OR "superior lateral genicular"[tiab] OR "inferior medial genicular"[tiab] OR "inferior lateral genicular"[tiab] OR "middle genicular"[tiab]) |
|  | #2 | ("knee osteoarthritis"[tiab] OR "knee OA"[tiab] OR "osteoarthritis of the knee"[tiab] OR "gonarthrosis"[tiab] OR "gonarthroses"[tiab] OR "knee pain"[tiab] OR "knee joint"[tiab] OR osteoarthriti*[tiab]) |
|  | #3 | (embolization[tiab] OR embolisation[tiab] OR emboliz*[tiab] OR embolis*[tiab] OR "transcatheter arterial"[tiab] OR transcatheter[tiab] OR "endovascular treatment"[tiab] OR "arterial embolization"[tiab] OR "arterial embolisation"[tiab] OR GAE[tiab] OR TAE[tiab]) |
|  | #4 | #1 AND #2 AND #3 |
|  | #5 | ("Arteries"[MeSH] OR "Knee Joint/blood supply"[MeSH]) |
|  | #6 | ("Osteoarthritis, Knee"[MeSH] OR "Osteoarthritis"[MeSH]) |
|  | #7 | ("Embolization, Therapeutic"[MeSH]) |
|  | #8 | #5 AND #6 AND #7 |
|  | #9 | #1 OR #5 |
|  | #10 | #2 OR #6 |
|  | #11 | #3 OR #7 |
|  | #12 | #9 AND #10 AND #11 |
| **Embase (Ovid)** |  |  |
|  | 1 | (genicular OR geniculate OR "genicular artery" OR "genicular arteries" OR "descending genicular" OR "superior medial genicular" OR "superior lateral genicular" OR "inferior medial genicular" OR "inferior lateral genicular" OR "middle genicular").ti,ab,kw. |
|  | 2 | ("knee osteoarthritis" OR "knee OA" OR "osteoarthritis of the knee" OR gonarthrosis OR gonarthroses OR "knee pain" OR osteoarthriti*).ti,ab,kw. |
|  | 3 | (embolization OR embolisation OR emboliz* OR embolis* OR "transcatheter arterial" OR transcatheter OR "endovascular treatment" OR "arterial embolization" OR "arterial embolisation" OR GAE OR TAE).ti,ab,kw. |
|  | 4 | 1 AND 2 AND 3 |
|  | 5 | exp artery/ OR exp knee/ |
|  | 6 | exp knee osteoarthritis/ OR exp osteoarthritis/ |
|  | 7 | exp artery embolization/ OR exp transcatheter embolization/ |
|  | 8 | 5 AND 6 AND 7 |
|  | 9 | 1 OR 5 |
|  | 10 | 2 OR 6 |
|  | 11 | 3 OR 7 |
|  | 12 | 9 AND 10 AND 11 |
| **Web of Science (Core Collection)** |  |  |
|  | #1 | TS=(genicular OR geniculate OR "genicular artery" OR "genicular arteries" OR "descending genicular" OR "superior medial genicular" OR "superior lateral genicular" OR "inferior medial genicular" OR "inferior lateral genicular" OR "middle genicular") |
|  | #2 | TS=("knee osteoarthritis" OR "knee OA" OR "osteoarthritis of the knee" OR gonarthrosis OR gonarthroses OR "knee pain" OR osteoarthriti*) |
|  | #3 | TS=(embolization OR embolisation OR emboliz* OR embolis* OR "transcatheter arterial" OR transcatheter OR "endovascular treatment" OR "arterial embolization" OR "arterial embolisation" OR GAE OR TAE) |
|  | #4 | #1 AND #2 AND #3 |
