# Supplementary material for: Outcome and Safety of Genicular Artery Embolization for Knee Osteoarthritis: A Systematic Review and Meta-Analysis
Source: Cardiovasc Intervent Radiol. 2026 Jun 16;49(9):1682–97. doi: 10.1007/s00270-026-04502-7 (PMC13597475; doi:10.1007/s00270-026-04502-7)
Supplement: Supplementary file 9 [file 270_2026_4502_MOESM7_ESM.docx]

| **Outcome(s)** | **Certainty** | **Plain language interpretation** | **Implication** |
| --- | --- | --- | --- |
| **KOOS-Pain (3, 6, 12 mo)** | **⬤⬤○○ Low** | GAE may improve KOOS-Pain scores by 20–25 points (exceeding MCID of 12). The 6-month estimate shows no heterogeneity (I² = 0%). Confidence is limited by the observational study designs and absence of blinding. | Further research is very likely to have an important impact and may change the estimate. |
| **WOMAC-Total (6 mo)** | **⬤⬤○○ Low** | GAE may reduce WOMAC-Total by approximately 30 points (exceeding MCID of 16.8) at 6 months, with moderate heterogeneity (I² = 60%). Prediction interval excludes zero. | Further research is very likely to have an important impact and may change the estimate. |
| **VAS (1, 3, 6, 12 mo)** | **⬤○○○ Very low** | The evidence suggests GAE may reduce VAS by 35–38 points (exceeding MCID of 20), but certainty is very low due to serious risk of bias, substantial heterogeneity (I² 85–90%), and suspected publication bias at 1 month. The true effect may be substantially different. | Any estimate of effect is very uncertain. Further research is needed before firm conclusions can be drawn. |
| **WOMAC-Total (12 mo)** | **⬤○○○ Very low** | The evidence is very uncertain. Only 3 studies contribute; prediction interval crosses zero ([−69.4; 15.9]), meaning some future studies may show no benefit. I² = 87% indicates major heterogeneity among the few available studies. | Any estimate of effect is very uncertain. Further research is needed before firm conclusions can be drawn. |
| **WOMAC-Pain** | **⬤○○○ Very low** | The evidence is very uncertain about the effect of GAE on WOMAC-Pain. Heterogeneity is substantial (I² 84–95%), and MCID for WOMAC-Pain subscale was not pre-specified. The 12-month prediction interval crosses zero. | Any estimate of effect is very uncertain. Further research is needed before firm conclusions can be drawn. |
| **Safety (AEs)** | **⬤○○○ Very low** | The evidence is very uncertain about the true rate of adverse events. Reported rates of skin discoloration (11%) and hematoma (3%) likely reflect inconsistent reporting definitions across studies rather than true variation in incidence. | Any estimate of effect is very uncertain. Further research is needed before firm conclusions can be drawn. |
