# Supplementary material for: Outcome and Safety of Genicular Artery Embolization for Knee Osteoarthritis: A Systematic Review and Meta-Analysis
Source: Cardiovasc Intervent Radiol. 2026 Jun 16;49(9):1682–97. doi: 10.1007/s00270-026-04502-7 (PMC13597475; doi:10.1007/s00270-026-04502-7)
Supplement: Supplementary file 10 [file 270_2026_4502_MOESM8_ESM.docx]

| **Study** | **D1 Randomization** | **D2 Deviations** | **D3 Missing data** | **D4 Measurement** | **D5 Reporting** | **Overall** |
| --- | --- | --- | --- | --- | --- | --- |
| **Bagla 2022** | Some concerns | **High** | Some concerns | Some concerns | Low | **High** |
| **Landers 2023** | Low | Some concerns | Low | Low | Low | Some concerns |
| **van Zadelhoff 2024** | Low | Some concerns | Low | Low | Low | Some concerns |
