# Supplementary material for: Outcome and Safety of Genicular Artery Embolization for Knee Osteoarthritis: A Systematic Review and Meta-Analysis
Source: Cardiovasc Intervent Radiol. 2026 Jun 16;49(9):1682–97. doi: 10.1007/s00270-026-04502-7 (PMC13597475; doi:10.1007/s00270-026-04502-7)
Supplement: Supplementary file 11 [file 270_2026_4502_MOESM9_ESM.docx]

| **Study** | **Domain** | **Judgment** | **Support for judgment** |
| --- | --- | --- | --- |
| **Bagla 2022** | D1 | Some concerns | Single-blinded (participants only); 2:1 allocation; small n (14 vs 7); allocation concealment not detailed. |
| **Bagla 2022** | D2 | **High** | 100% sham crossover at 1 month; no control data beyond 1 month; comparison valid only at 1-month timepoint. |
| **Bagla 2022** | D3 | Some concerns | 5 subjects removed post-crossover; 16/21 analyzed at 12 months. |
| **Bagla 2022** | D4 | Some concerns | Single-blind; self-reported PROMs (VAS, WOMAC); sham = arteriography without embolization. |
| **Bagla 2022** | D5 | Low | Pre-registered NCT03362957; FDA IDE; outcomes aligned with protocol. |
| **Landers 2023** | D1 | Low | Triple-blinded; 1:1 allocation; groups balanced at baseline. |
| **Landers 2023** | D2 | Some concerns | Protocol changed mid-trial: first 4 patients single-vessel, then multi-vessel embolization; sham 30–60 min groin access. |
| **Landers 2023** | D3 | Low | 58/59 completed 12-month questionnaires (1 TKA dropout); ITT analysis. |
| **Landers 2023** | D4 | Low | Triple-blinded; validated KOOS; independent blinded assessor. |
| **Landers 2023** | D5 | Low | Pre-registered ACTRN12616001184460; primary outcome (KOOS-Pain) reported per protocol. |
| **van Zadelhoff 2024** | D1 | Low | Double-blind sham-controlled; 1:1 concealed allocation; recruited from 5 hospitals. |
| **van Zadelhoff 2024** | D2 | Some concerns | 3/29 GAE patients had no blush and were not embolized (effectively received sham); all procedures by single IR. |
| **van Zadelhoff 2024** | D3 | Low | 0 dropouts; 58/58 analyzed; ITT. |
| **van Zadelhoff 2024** | D4 | Low | Double-blind; validated KOOS; blinded assessors. |
| **van Zadelhoff 2024** | D5 | Low | Pre-registered NCT03884049; outcomes aligned with protocol. |
